# Investigation of the Effects of Working Conditions on Performance of Planar and Cylindrical Proton Exchange Membrane Fuel Cells

**DOI:** 10.1002/gch2.202300096

**Published:** 2023-08-18

**Authors:** Muhammed Salih Cellek, Tolga Demircan

**Affiliations:** ^1^ Department of Mechanical Engineering Graduate School of Natural and Applied Sciences Kirikkale University Kirikkale 71450 Turkey; ^2^ Department of Mechanical Engineering Faculty of Engineering and Architecture Kirikkale University Kirikkale 71450 Turkey

**Keywords:** cylindrical fuel cell, PEM fuel cell, planar fuel cell, proton exchange membrane fuel cell

## Abstract

In this study, the effects of varying operating conditions on cell performance in cylindrical and planar PEM (Proton Exchange Membrane) fuel cells with equal geometrical properties are investigated numerically. For this purpose, planar and cylindrical fuel cells are analyzed and the changes in performance are compared with each other. In particular, by increasing the temperature from 363 to 373 K, the current density decreases by ≈50%. When the pressure is increased from 1 atm to 1.2 atm, the current density increases by 20% and 2% in cylindrical and planar fuel cells, respectively. When the stoichiometric ratio is increased from 1 to 1.5, the current density increases by 25% and 32% for cylindrical and planar fuel cells, respectively. However, it decreases slightly after 1.5 value of the stoichiometric ratio. As a result, it is seen that temperature has a more significant effect on fuel cell performance than other operating conditions. It can be said that the planar fuel cell can operate with more stable performance than the cylindrical fuel cell when the temperature and pressure are changed. However, in the case of changing the stoichiometric ratio, there is no significant difference in the operating performance of both fuel cells.

## Graphical abstract

In this study, the effects of varying operating conditions on cell performance in cylindrical and planar PEM (Proton Exchange Membrane) fuel cells with equal geometrical properties are investigated numerically. The effects of varying operating conditions on the performance of planar and cylindrical fuel cells are analyzed and the changes in performance are compared with each other.

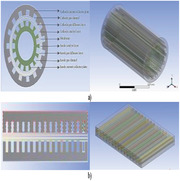

## 1 Introduction

The increase in population and the development of technology increase the demand for energy in the world day by day. The studies carried out by scientists to meet this increasing demand are of great importance. Today, most of the energy demands are met by energy obtained from fossil resources. Fossil resources have disadvantages such as having limited reserves and the emission resulting from their combustion being harmful to human health and the environment. Therefore, in order to meet the increasing energy demand, studies on renewable energy sources, which are characterized as unlimited, sustainable, and clean energy sources, have been increased by scientists. The main renewable energy sources can be listed as solar, wind, geothermal, biomass, wave, hydrogen energy, etc.

PEM fuel cell is a power generator that converts hydrogen and oxygen into electrical energy through an electrochemical reaction. Thermodynamic efficiency is up to %60. This rate is ≈30% in engines operating with fossil fuels, which are called conventional engines^[^ ^1^ ^]^. PEMFC (Proton Exchange Membrane Fuel Cell)stands out among other fuel cells due to its high efficiency, low operating temperature, easy activation, and applicability.^[^ ^2^ ^]^ However, further improvements are needed for the PEM fuel cell to be commercially viable. The design of PEMFC is one of these improvements.^[^ ^3^ ^]^ In the literature, there are many studies investigating the effect of fuel cell design on fuel cell performance.

When these studies are reviewed, it is seen that one of the most important factors affecting fuel cell performance is the design of the flow channels that ensure that the reactant gases are evenly distributed throughout the cell and that excess water is effectively discharged. In this context, some studies in the literature are mentioned below.

Dhahad et al.^[^ ^4^ ^]^ experimentally investigated the effect of channel geometry on fuel cell performance by designing eight different flow channel: parallel‐z, serpentine‐z, serpentine‐2z, serpentine‐3z, serpentine‐s, serpentine‐2s, serpentine‐w, and novel serpentine‐modified w. They obtained the best performance in the fuel cell using novel serpentine‐modified w flow channel, which provides uniform distribution of reactant gases throughout the cell. Jeon et al.^[^ ^5^ ^]^ numerically investigated the effect of serpentine flow channel designs on PEM fuel cell performance. In this scope, they designed the flow channels as single, double, cyclic‐single, and symmetric‐single. In the study, they used high and low inlet humidity as varying operation condition. They obtained the highest performance value and more uniform current density distribution in the double channel design at high inlet humidity. However, at low inlet humidity, the performance values for four different flow channels were close to each other. They also stated that at low inlet humidity, the pressure drop is less in the cyclic‐single and symmetric‐single flow channel and therefore it may be an advantage to use these types of flow channels in larger‐scale fuel cells.

Muthukumar et al.^[^ ^6^ ^]^ have examined the effect of flow channel cross‐sectional area on fuel cell performance by designing flow channel geometries with 0.5×0.5, 1×1, 1.5×1.5, and 2×2 as width and height dimensions provided that the channel length remains constant. As a result of the study, they concluded that the power and current density values generated in the fuel cell with 0.5×0.5 dimensions are higher the other channel geometries due to better water management although the active area is smaller. Shimpalee and Zee^[^ ^7^ ^]^ numerically investigated the effect of the relationship between flow channel width and rib width on fuel cell performance. For this purpose, they used three flow field configurations without changing the fuel cell total area. This study revealed that the fuel cell with a narrow flow channel and wide rib width has higher performance. They also obtained that the current density is not distributed evenly throughout the cell in a wide flow channel and narrow rib width and the heat transfer is better with increasing rib width. Shimpalee et al.^[^ ^8^ ^]^ investigated the effect of flow channel length on cell performance in another study. As a conclusion of the study, it was concluded that short flow channel length provides a more uniform current density distribution and also causes less condensed water, which has a positive effect on the fuel cell performance. They also stated that the length of the flow channel is a major factor for the performance, efficiency, and durability of the fuel cell. In addition, in the study conducted by Carcadea et al.^[^ ^9^ ^]^ it was revealed that cell performance increased with decreasing cathode flow channel dimensions due to the removal of water from the cell being easier with increasing velocity. Zhou et al.^[^ ^10^ ^]^ conducted a study comparing the performance of a fuel cell with a sinusoidal flow field and a fuel cell with a parallel flow field. As a result of the study, the fuel cell with a sinusoidal flow field showed higher performance compared to the fuel cell with a parallel flow channel due to favorable effects of mass transfer capacity and water removal performance.

In the literature, there are many studies examining the effect of changing operating conditions on fuel cell performance. Ozdogan et al.^[^ ^11^ ^]^ numerically investigated the effect of operating pressure and temperature on PEM fuel cell performance. In their study, non‐isothermal, steady‐state, and single‐phase models were used. Analyses were carried out at three different temperatures and three different pressure values, keeping the humidity constant. The results showed that the cell performance increases as the temperature value increases, the pressure does not have a great effect on the cell performance, however, the effect of temperature on performance is more apparent at high pressures. Cellek and Bilgili^[^ ^12^ ^]^ conducted a numerical study to investigate the effect of stoichiometry ratio on fuel cell performance. For this purpose, numerical analyses were carried out for five different voltages and five different stoichiometry ratios in a two‐cell PEM fuel cell stack. They stated that as the stoichiometric ratio increases, the current density increases but the rate of increase decreases, reactant gases are more evenly distributed and the membrane temperature increases slightly. Yan et al.^[^ ^13^ ^]^ experimentally investigated the effect of humidity, operating temperature, and pressure on fuel cell performance. They reported that the decrease in the cathode humidity ratio negatively affects the fuel cell performance. They also observed that the change in temperature has a significant effect on membrane conductivity and water transport in the gas diffusion layer and catalyst layer and the stable and dynamic structure of the fuel cell improves with increasing pressure.

In recent years, scientists have been studying new fuel cell designs that can be an alternative to the planar fuel cell called conventional design. The cylindrical design stands out as an alternative to the conventional design due to its low production costs, good water management, high volumetric, and gravimetric properties.^[^ ^14^ ^]^ Sierra et al.^[^ ^15^ ^]^ applied the flow channel designs commonly used in the literature to a cylindrical fuel cell and conducted numerical analyses under various operating conditions. The results of the analyses were compared with a planar fuel cell having the same active area in the literature. The study revealed that the cylindrical fuel cell has more uniform pressure, current density, and reactant gases distribution compared to the planar fuel cell. Therefore, they stated that it can be used as an alternative to planar fuel cell especially in portable applications. In another study comparing the performance of cylindrical fuel cell and planar fuel cell; it has been concluded that higher current density is obtained and reactant gases, water, and temperature are more evenly distributed in the cylindrical fuel cell.^[^ ^16^ ^]^ Bullecks et al.^[^ ^17^ ^]^ experimentally investigated cylindrical and planar fuel cells and compared them in terms of performance. As a result, the authors determined that the cylindrical fuel cell has better performance in high‐voltage regions, but has higher ohmic resistance compared to the planar fuel cell. They also stated that the cylindrical fuel cell can be used especially in portable applications due to its higher volumetric and gravimetric power density compared to the planar fuel cell. Gunduz and Demircan^[^ ^18^ ^]^ investigated the effect of current collector plate geometry on cylindrical fuel cell performance. For this purpose, they designed four different anode and cathode flow channels as straight‐straight, helix‐helix, straight‐helix, and helix‐straight. Numerical analyses were conducted for each design at three different pressure, four different flow rates, and ten different voltages. As a result of the analyses, it was concluded that the current density and pressure difference values in the fuel cell with helix flow channel design are higher than the fuel cell with straight flow channel design; however, the power density values do not change much.

The studies in the literature on PEM fuel cells are generally focused on fuel cells with planar design. Although many studies have been carried out on fuel cells, problems such as cost and water management have not been fully solved. Therefore, novel fuel cell geometries that can be an alternative to the planar fuel cell have been designed recently. Cylindrical fuel cells are one of the alternative geometries. There are studies in the literature that the performance of cylindrical fuel cells and planar fuel cells are close to each other. However, there are limited studies comparing the performances of cylindrical and planar PEM fuel cells under varying operating conditions and examining the responses of fuel cells to changing conditions in terms of performance. Therefore, in this study, it is aimed to investigate the performances of cylindrical and planar PEM fuel cells under varying conditions and to compare the changes in their performances with each other. In studies comparing cylindrical and planar fuel cell performances, it has been observed that only the active area of both fuel cells is taken as the same. Since there are many design parameters that affect the performance of fuel cells, it is foreseen that only keeping the active area constant in the comparison of two fuel cells and accepting two cells as equivalent may cause faults. For this reason, unlike the studies in the literature, in this study, both fuel cells were designed based on the same design criteria. Thus it is aimed to make a more reliable comparison. In this context, planar and cylindrical fuel cells with the same active area, flow channel length and width, current collector plate thickness, gas diffusion layer thickness, membrane thickness, and catalyst thickness were designed. For these two fuel cells, numerical analyses were carried out at five different temperatures, six different pressures, and six different stoichiometric ratios.

## 2 Model Definition and Mathematical Formulation

In this study, the performances of cylindrical and planar PEM fuel cells under different operating conditions were investigated. As the cylindrical PEM fuel cell reference geometry, the design in the article of Sierra et al.^[^ ^15^ ^]^ was preferred. For a proper comparison, a planar fuel cell with the same active area, channel length and width, flow contact area, and membrane‐electrode assembly thickness as the cylindrical fuel cell in the reference geometry was designed. The geometrical and electrochemical properties of the cylindrical and planar fuel cell are shown in **Table** **1**. By using these fuel cell geometries, numerical analyses were conducted for different operating parameters. As a result of the analysis, the performances of cylindrical and planar fuel cells under varying operating conditions and their reactions to parameter variation were comparatively analyzed.

**Table 1:** Geometrical and electrochemical properties

Parameter	Value
Gas channel width [mm]	0.8
Gas channel height [mm]	0.8
Gas channel length [mm]	20
Gas channel number [mm]	15
Fuel cell length [mm]	20
Average radius [mm]	4.37
Active area [mm^2^]	549
Plate thickness [mm]	1.6
Gas diffusion layer thickness [mm]	0.25
Catalyst thickness [mm]	0.02
Membrane thickness [mm]	0.178
Anode reference exchange current density [A cm^−2^]	0.2
Cathode reference exchange current density [A cm^−2^]	1×10^−4^
Anode charge transfer coefficient	2
Cathode charge transfer coefficient	2
Anode concentration exponent	0.5
Cathode concentration exponent	1
Open circuit voltage [V]	0.95
Gas diffusion layer porosity	0.4
Catalyst layer porosity	0.2
Membrane equivalent weight [kg kmol^−1^]	1100
Gas diffusion layer viscous resistance [m^−2^]	1×10^12^
Catalyst layer viscous resistance [m^−2^]	1×10^12^

**Figure 1:**
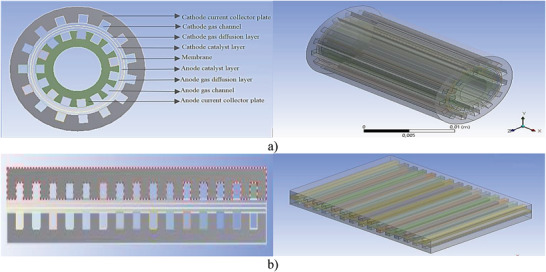
Front and isometric views of geometry a) cylindrical PEM fuel cell b) planar PEM fuel cell.

The geometry and component names for the cylindrical and planar PEM fuel cells designed in this study have been provided in **Figure** **1**. As the figure shows, PEM fuel cells consist of nine different parts from the outside to the inside of fuel cell: the cathode current collector plate, cathode gas channel, cathode gas diffusion layer, cathode catalyst layer, membrane, anode catalyst layer, anode gas diffusion layer, anode gas channel, and anode current collector plate, respectively. The physical properties of the PEM fuel cell layers^[^ ^19^ ^]^ and the thermophysical properties of the fluids^[^ ^19^ ^]^ were selected in accordance with the literature and shown in **Tables** **2** and **3**, respectively.

**Table 2:** The physical properties of the layers.^[^ ^19^ ^]^

Physical Property	Current Collector Plate	Gas Diffuser Plate	Catalyst Plate	Membrane
Thermal conductivity [W m^−1^ K^−1^]	20	1674	70	0,24
Electricity conductivity [ohm m]^−1^	10^4^	1250	1250	1×10^−16^
Surface/Volume Ratio [m^−1^]	–	–	200 000	–

**Table 3:** Thermophysical properties of the fluids.^[^ ^19^ ^]^

Physical Property	H_2_	O_2_	H_2_O (water vapor)
Specific heat [J kg^−1^ K^−1^]	14283	919.31	2014
Thermal conductivity [W m^−1^ s^−1^]	0.1672	0.0246	0.0261
Viscosity [kg m^−1^ s^−1^]	8.411×10^−6^	1.919 × 10^−5^	1.34 × 10^−5^
Molecular weight	2.01594	31.9988	18.01534
Electricity conductivity [ohm m]^−1^	1 × 10^−16^	1 × 10^−16^	1 × 10^−16^

### 2.1 Model Assumptions

The numerical analyses are conducted based on some assumptions, which are given below;

a) The fuel cells operate steady state.
b) The reactant gases and products in the fuel cells show ideal gas behavior.
c) The gas flow in the gas channels is incompressible and laminar.
d) The water released as a result of the electrochemical reactions is only the vapor phase.
e) The membrane is completely damp and impermeable to the reactants.
f) Lifting force effects have been neglected.

### 2.2 Differential Equations

The general form of the conservation of mass, momentum, energy, species, and charge transfer equations to be used in the numerical analyses of planar and cylindrical PEM fuel cells are given below.^[^ ^11^, ^15^, ^20^ ^]^

Conservation of mass

(1) $$\nabla .\left(\rho \vec{v}\right)\;=\;S_{m}$$

ρ, $\vec{v}$, and S_m_ refer to density of the gas mixture, vector velocity, and source term, respectively.

Conservation of momentum

(2) $$\nabla .\;\left(\rho \vec{v}\vec{v}\right)=\;-\nabla p+\nabla \cdot \left(\mu^{eff}\nabla \vec{v}\right)+S_{p}$$

p, µ^*eff*^, and S_p_ terms in this equation define static pressure, average viscosity of the mixture, and source term, respectively.

Species transport

(3) $$\nabla \cdot \;\left(\varepsilon uC_{k}\right)=\;\nabla .\left(D_{k}^{eff}\nabla C_{k}\right)+S_{k}$$

In the equation, S_k_ is the source term and $D_{k}^{\mathrm{eff}}$ is the effective gas species diffusivity coefficient which is determined by means of Equation 4.

(4) $$D_{k}^{eff}=\;\varepsilon^{1.5}D_{k}$$

Conservation of energy

(5) $$\nabla .\;\left(\varepsilon \rho \vec{u}h\right)=\;\nabla .\left(k\nabla T\right)+S_{h}$$

S_h_, h, k, and T terms in this equation represent energy source term, enthalpy, thermal conductivity, and temperature, respectively.

### 2.3 Boundary Conditions

In this study, anode and cathode mass flow rate is taken as inlet boundary condition and outlet pressure is taken as outlet boundary condition. In addition, a constant temperature boundary condition was defined for the anode and cathode terminal surfaces, and variable voltage values (0.4 V, 0.5 V, 0.6 V, 0.7 V, 0.8 V) were used for the cathode.

In the first section of the analyses where the effect of temperature was examined, the operating pressure was kept constant as 2 atm and analyses were carried out in cylindrical and planar PEM fuel cells for five different temperature values (333 K, 343 K, 353 K, 363 K, 373 K). In these analyses, the anode stoichiometric ratio and the cathode stoichiometric ratio were taken as 1.25 and 2, respectively. In the second section of the analyses where the effect of pressure was examined, the temperature value was kept constant at 343 K, and analyses were conducted at 6 different operating pressure values (1 atm, 1.2 atm, 1.4 atm, 1.6 atm, 1.8 atm, 2 atm). Anode and cathode stoichiometric ratio were based on the values used in the first section analyses. In the third section analyses, where the effect of the stoichiometric ratio was investigated, six different stoichiometric ratios (1, 1.5, 2, 2.5, 3, 3.5) were determined and analyses were carried out by taking 343 K and 2 atm as constant, where the highest current densities were obtained in the previous two sections. The relative humidity of the anode side was taken as 50% and the relative humidity of the cathode side was taken as 100% in all of the analyses conducted. While determining the operating parameters used in the analyses, frequently used values in the literature were taken into account. The operating conditions used in the first, second, and third parts of the analyses are shown in **Table** **4** as a summary. The boundary conditions used in this study are given in **Table** **5**.

**Table 4:** Operating conditions for first, second, and third sections of the analysis

Parameters	Section I	Section II	Section III
Pressure [atm]	2	1, 1.2, 1.4, 1.6, 1.8, 2	2
Anode stoichiometric ratio	1.25	1.25	1, 1.5, 2, 2.5, 3, 3.5
Cathode stoichiometric ratio	2	2	1, 1.5, 2, 2.5, 3, 3.5
Anode relative humidity	50%	50%	50%
Cathode relative humidity	100%	100%	100%
Temperature [K]	333, 343, 353, 363, 373	343	343

**Table 5:** Boundary conditions of fuel cell

Location in the fuel cell geometry	Type of boundary condition
Anode channel inlet	$\;\dot{m}$=$\dot{m}\;$_in_, T = T_in_, v = 0, w = 0, $C_{H_{2}}$= $C_{H_{2},in}^{a}$, $C_{H_{2}O}\;$= $C_{H_{2}O,in}^{a}$
Cathode channel inlet	$\;\dot{m}$=$\dot{m}\;$_in,_ T = T_in_, v = 0, w = 0, $C_{O_{2}}$= $C_{O_{2},in}^{c}$, $C_{N_{2}}\;$= $C_{N_{2},in}^{c}$
Anode and cathode gas channel outlet	$\;\frac{\partial u}{\partial x}=\;\;\frac{\partial v}{\partial x}=\;\;\frac{\partial w}{\partial z}=\;\;\frac{\partial T}{\partial x}=\;0$
Interface of catalyst and membrane	u = v = w = C_i_ = 0
Upper surface of gas channels	u = v = w = C_i_ = 0
Lower surface of gas channels	u = v = w = 0

### 2.4 Numerical Solution

In this study, the continuity, momentum, energy, species, and electrochemical equations were solved numerically with the help of Computational Fluid Dynamics by using defined boundary conditions. For this purpose, the Fluent package program was used. The standard method was applied to discretize the pressure equations and the First Order Upwind method was applied to discretize the other equations. In this context, SIMPLE solution algorithm was preferred for solving the discretized equations.

**Figure 2:**
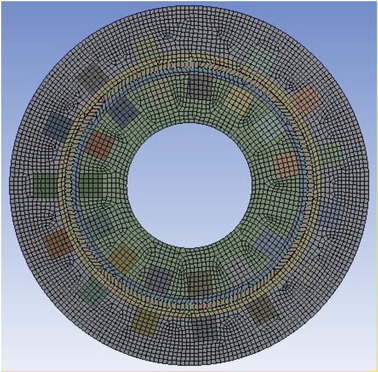
Mesh structure.

The mesh structure used in the numerical analysis can affect the results obtained. For this reason, in this study, mesh independence was performed. For this purpose, mesh structures with different numbers of elements were created. Analyses were repeated for these mesh structures. Optimum mesh structures were determined according to the results obtained. In this context, the mesh structure with 724 052 elements for the cylindrical fuel cell and the mesh structure with 721 259 elements for the planar fuel cell were selected as the optimum mesh structure. These optimum mesh structures were used in all simulations performed in this study. An example of the mesh structure for the cylindrical fuel cell is given in **Figure** **2**.

**Figure 3:**
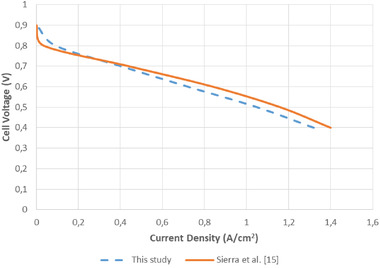
Comparison with literature results with the study of Sierra et al.^[^ ^15^ ^]^

In order to test the accuracy of the numerical method to be used in the analyses and the results to be obtained as a result of the analyses, comparison was made with a similar study in the literature. The numerical study carried out by Sierra et al.^[^ ^15^ ^]^ for a cylindrical fuel cell is taken as a reference for validation. In order to be able to compare the obtained results properly, numerical analyses were conducted by taking the geometry, operating parameters, and boundary conditions in the reference study exactly the same. The comparison of the polarization curve obtained as a result of the numerical analyses and the polarization curve obtained as a result of the experimental studies of Sierra et al. is given in **Figure** **3**. As can be seen from the figure, the current density values obtained as a result of numerical and experimental analyses are quite close to each other. When the polarization curves are analyzed, it is seen that the results obtained in this study and the results of the literature^[^ ^15^ ^]^ are largely compatible with each other. Therefore, it is possible that the numerical method used in the numerical analyses conducted during this study and the reliability of the results obtained are at an acceptable level.

## 3 Results and Discussion

In this study, the performance of planar and cylindrical PEM fuel cells under varying operating conditions was investigated comparatively. In order to a proper comparison, planar and cylindrical fuel cells with equivalent active areas, flow channel sizes, contact surface areas, and membrane electrode assembly thicknesses were designed. In addition, the physical properties of the fuel cell components were chosen to be the same for both fuel cells. The cathode voltage value was taken as a variable in the analyses. In the first stage, the performances of the planar and cylindrical fuel cell were compared under constant operating conditions (2 atm and 343 K). Then, numerical analyses were conducted for both fuel cells under varying operating conditions. The analyses were carried out for fuel cell models at five different temperature values at constant pressure, six different pressure values at constant temperature, and six different stoichiometric ratio values at constant temperature and pressure. The results obtained in these analyses are given and discussed in the section below.

### 3.1 The Effect of Fuel Cell Design on Fuel Cell Performance

**Figure 4:**
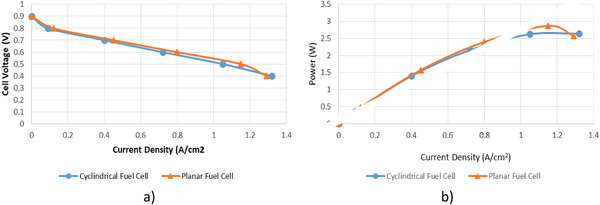
Comparison of cylindrical and planar PEM fuel cell.

Numerical analyses were carried out for five different cathode voltage values, taking operating conditions constant for both fuel cells as 2 atm, 343 K, anode stoichiometric ratio of 1.25, and cathode stoichiometric ratio of 2. The polarization curves showing the current density‐voltage values obtained as a result of the analyses are shown in **Figure** **4**a. The figure shows that the cylindrical and planar fuel cells have similar polarization curves. When the current density values obtained in both fuel cells are compared, it is seen that the maximum current density difference was 0.1 A cm^−2^ at 0.5 V and the minimum current density difference was 0.03 A cm^−2^ at 0.4 and 0.8 V.

The power‐current density curve of the fuel cells obtained as a result of the analyses is shown in Figure 4b. The maximum power generated in the cylindrical fuel cell was 2.64 W at 0.4 V, while in the planar fuel cell, it was 2.875 W at 0.5 V. When the power‐current density curve is analyzed, it is observed that both fuel cells have a similar profile and the curves get closer to each other at low current density values. In addition, it was observed that the power generated in the planar fuel cell was slightly higher than the cylindrical fuel cell at high current density values.

### 3.2 The Effect of Temperature on PEM Fuel Cell Performance

**Figure 5:**
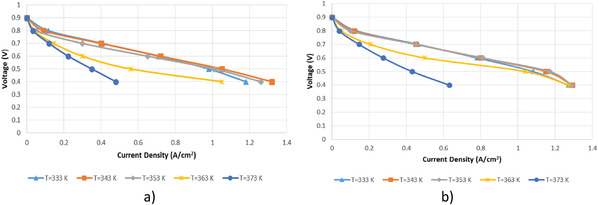
Polarization curve for different temperatures a) cylindrical PEM fuel cell b) planar PEM fuel cell.

The constant operating conditions for both fuel cells were determined as 2 atm, anode stoichiometric ratio of 1.25, cathode stoichiometric ratio of 2. The analyses were carried out at 333 K, 343 K, 353 K, 363 K vs 373 K at five different voltage values. The polarization curves obtained as a result of the analyses are given in **Figure** **5**. When the polarization curve of the cylindrical fuel cell shown in Figure 5a is examined, it is seen that the highest current density is 1.32 A cm^−2^ at 343 K and 0.4 V. However, the highest current density values at varying voltage values were reached at 343 K. At varying voltage values, the highest current density values were reached at 343 K, and at varying temperature values, the highest current density values were reached at 0.4 V. When the current density values at 0.4 V are compared, it is observed that 12% increment between 333 K and 343 K, 5% decrement between 343 K and 353 K, 17% decrement between 353 K and 363 K, and 54% decrement between 363 K and 373 K.

When the polarization curve of the planar fuel cell shown in Figure 5b is examined, it is seen that the highest current density value is 1.29 A cm^−2^ at 343 K and 353 K at 0.4 V. In addition, the highest current density values were obtained at 343 K and 353 K at varying voltage values.

The rate of change in current density at 0.4 V and varying temperatures is given in given in **Table** 6 for cylindrical fuel cell and planar fuel cell. As can be seen the table, when the temperature is changed in the range of 333 K – 363 K in the planar fuel cell, there is no significant change in the current density value while the current density value decreases in the cylindrical fuel cell. Therefore, it is observed that the planar fuel cell works more stable in the 333 K and 363 K than the cylindrical fuel cell. However, when the temperature is increased from 363 K to 373 K, there is a decrease of ≈50% in the current density value in both fuel cells.

**Table 6:** Current density variation with temperature change at 0.4 V

Temperature Variation	Cylindrical Fuel Cell		Planar Fuel Cell	
	Variance [%]	Trend	Variance [%]	Trend
333–343 K	12	Increasing	2	Increasing
343–353 K	5	Decreasing	0	–
353–363 K	17	Decreasing	1	Decreasing
363–373 K	54	Decreasing	51	Decreasing

### 3.3 The Effect of Pressure on PEM Fuel Cell Performance

**Figure 6:**
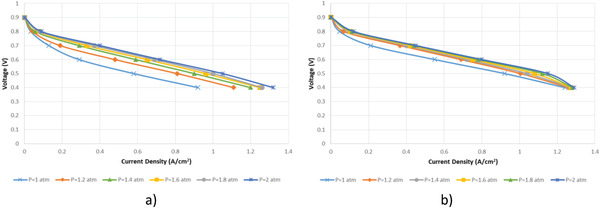
Polarization curve for different pressures a) cylindrical PEM fuel cell b) planar PEM fuel cell.

In the analysis, pressure values of 1 atm, 1.2 atm, 1.4 atm, 1.6 atm, 1.8 atm, and 2 atm were defined as varying operating conditions. In addition, anode stoichiometric ratio and cathode stoichiometric ratio of 1.25 and 2, respectively, and 343 K were defined as constant conditions, and the analyses were carried out at five different voltage values. The polarization curves obtained as a result of the analyses are shown in **Figure** **6**. When the polarization curves obtained were examined, it was observed that the current density increases with increasing pressure in both fuel cells, but the rate of the increase slows down in general. It was also observed that the current density value of the cylindrical fuel cell increased by 21% between 1 atm and 1.2 atm, 8% between 1.2 atm and 1.4 atm, 4% between 1.4 atm and 1.6 atm, 0.8% between 1.6 atm and 1.8 atm, and 5% between 1.8 atm and 2 atm.

When the current density values obtained at varying pressure values at 0.4 V for the planar fuel cell are compared, it is seen that there is an increase of 2% between 1 atm and 1.2 atm, 0.8% between 1.2 atm and 1.4 atm, 0.24% between 1.4 atm and 1.6 atm, 0.55% between 1.6 atm and 1.8 atm, and 0.78% between 1.8 atm and 2 atm.

The rate of change in current density at varying pressure values is given in **Table** 7 for both fuel cells. The rate of increase in current density with increasing pressure in the cylindrical fuel cell is higher than the rate of increase in the planar fuel cell. Therefore, it is observed that pressure variation is more effective on current density in the cylindrical fuel cell than in the planar fuel cell. As a result, it is determined that the planar fuel cell can work more stable than the cylindrical fuel cell in the change of pressure.

**Table 7:** Current density variation with pressure change at 0.4 V

Pressure variation	Cylindrical fuel cell		Planar Fuel Cell	
	Variance [%]	Trend	Variance [%]	Trend
1.0 atm–1.2 atm	21	Increasing	2	Increasing
1.2 atm–1.4 atm	8	Increasing	0.8	Increasing
1.4 atm–1.6 atm	4	Increasing	0.24	Increasing
1.6 atm–1.8 atm	0.8	Increasing	0.55	Increasing
1.8 atm–2.0 atm	5	Increasing	0.78	Increasing

### 3.4 The Effect of Stoichiometric Ratio on PEM Fuel Cell Performance

**Figure 7:**
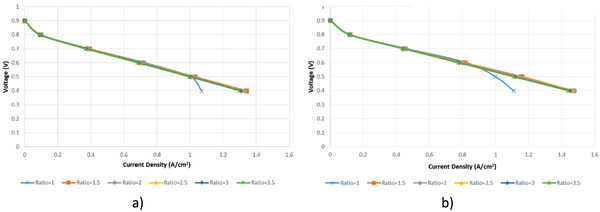
Polarization curve for different stoichiometric ratios a) cylindrical PEM fuel cell b) planar PEM fuel cell.

In this section, the effect of varying the stoichiometric ratio on PEM fuel cell performance is analyzed. In this context, analyzed were conducted for cylindrical and planar fuel cells at constant 2 atm and 343 K at 5 different voltage values by changing the anode and cathode stoichiometric ratio as 1, 1.5, 2, 2.5, 3, and 3.5. The polarization curve obtained for the cylindrical fuel cell is given in **Figure** **7**a and for planar fuel cell in Figure 7b. When the polarization curves of both fuel cells are examined, current density increases as the voltage values decrease for all stoichiometric ratios. For the cylindrical fuel cell, the highest current density value was obtained at a stoichiometric ratio of 1.5 and a voltage value of 0.4, while for the planar fuel cell, the highest current density value was obtained at a stoichiometric ratio 2 and a voltage value of 0.4. For both fuel cells, the curves plotted for all stoichiometric ratios at high cell voltage values are similar each other. However, for stoichiometric ratio 1, when the cell voltage is below 0.5 V in the cylindrical fuel cell, the current density value shows a significant decrease compared to the current density value obtained for other stoichiometric ratios. A similar situation is observed in the planar fuel cell with a stoichiometric ratio of 1 and a cell voltage of 0.6.

At varying stoichiometric ratios, the rate of change in current density for the case where the cell voltage value is 0.4 V is given together in **Table** 8 for both fuel cells. As can be seen from the table, a significant increase in current density is observed when the stoichiometric ratio is increased from 1 to 1.5 that is 25% in the cylindrical fuel cell and 32% in the planar fuel cell. As the stoichiometric ratio is increased above 1.5, slight decreases in current density are observed, which is similar for cylindrical and planar fuel cells.

**Table 8:** Current density variation with stoichiometric ratio change at 0.4 V

Stoichiometric ratio variation	Cylindrical fuel cell		Planar fuel cell	
	Variance [%]	Trend	Variance [%]	Trend
1.0–1.5	25	Increasing	32	Increasing
1.5–2.0	1	Decreasing	0.4	Increasing
2.0–2.5	0.8	Decreasing	0.8	Decreasing
2.5–3.0	0.7	Decreasing	0.8	Decreasing
3.0–3.5	0.53	Decreasing	0.7	Decreasing

## 4 Conclusions

In this study, the performances of cylindrical and planar fuel cells under varying operating conditions were investigated comparatively. In the first part of this study, cylindrical and planar fuel cells with equivalent geometrical dimensions and electrochemical properties were designed and analyzed under the same operating conditions. As a result of the analysis, it was observed that the planar and cylindrical fuel cell have a similar polarization curve and the power‐current density curves overlap largely. In the second part of the study, the effect of various operating parameters on the performance on the performance of cylindrical and planar PEM fuel cells was investigated and the reactions of both fuel cells to varying operating conditions were observed. The results of the analyses show that temperature is a factor that significantly affects the current density for both fuel cells. In addition, the effect of pressure and stoichiometric ratio on the performance was seen to be less compared to the effect of temperature factor on the performance. It was also observed that the current density values obtained in the cylindrical fuel cell varied more than the current density values obtained in the planar fuel cell. As a result of the analyses, although the current density values obtained from the cylindrical and planar fuel cell under appropriate operating conditions are close to each other, when the reacts to changing operating conditions at the same voltage value are analyzed in terms of fuel cell performance, it is concluded that the planar fuel cell has less change and provides more stable current density compared to the cylindrical fuel cell under changing operating conditions.

## Conflict of Interest

The authors declare no conflict of interest.

## Data Availability

The data that support the findings of this study are available from the corresponding author upon reasonable request.
